# Rating and Ranking the Role of Bibliometrics and Webometrics in Nursing and Midwifery

**DOI:** 10.1155/2014/135812

**Published:** 2014-01-06

**Authors:** Patricia M. Davidson, Phillip J. Newton, Caleb Ferguson, John Daly, Doug Elliott, Caroline Homer, Christine Duffield, Debra Jackson

**Affiliations:** ^1^Johns Hopkins University (JHU), Baltimore, MD 21218, USA; ^2^University of Technology, Sydney (UTS), Sydney, NSW 2007, Australia

## Abstract

*Background*. Bibliometrics are an essential aspect of measuring academic and organizational performance. *Aim*. This review seeks to describe methods for measuring bibliometrics, identify the strengths and limitations of methodologies, outline strategies for interpretation, summarise evaluation of nursing and midwifery performance, identify implications for metric of evaluation, and specify the implications for nursing and midwifery and implications of social networking for bibliometrics and measures of individual performance. *Method*. A review of electronic databases CINAHL, Medline, and Scopus was undertaken using search terms such as bibliometrics, nursing, and midwifery. The reference lists of retrieved articles and Internet sources and social media platforms were also examined. *Results*. A number of well-established, formal ways of assessment have been identified, including *h*- and *c*-indices. Changes in publication practices and the use of the Internet have challenged traditional metrics of influence. Moreover, measuring impact beyond citation metrics is an increasing focus, with social media representing newer ways of establishing performance and impact. *Conclusions*. Even though a number of measures exist, no single bibliometric measure is perfect. Therefore, multiple approaches to evaluation are recommended. However, bibliometric approaches should not be the only measures upon which academic and scholarly performance are evaluated.

## 1. Introduction

Increasingly, individual researchers and academic institutions are being required to rate and rank publications as a metric of both individual researcher and organisational performance [1, 2]. This trend is international, and while bibliometrics are not new, increased surveillance on the outputs from academic sectors through evaluation measures such as Excellence in Research for Australia (ERA), Research Assessment Exercise (RAE) in the United Kingdom, and Performance-Based Research Fund (PBRF) in New Zealand have spurred interest and attention of academic nurses and midwives to ensure that these metrics adequately represent the quality of the research [3].

The term “bibliometrics” describes a mathematical method for counting the number of academic publications and related citations and is based on authorship. Measures such as citations, impact factors (IFs), *h*- and *c*-indices are commonly calculated. Data to inform a bibliometric analysis can be extracted from a range of online databases such as Thompson Reuters Web of Science or Elsevier-Scopus.

Bibliometrics are one way used to measure the impact of the research although the notion of impact is often difficult to measure. Another common criticism is that historical approaches to bibliometrics, such as impact factors and citations, disadvantage some disciplines. Reasons for this are complex including the often competing demands of a practice-based discipline, whereby published research findings may be measured in changed and improved clinical practice rather than citations [4, 5]. In addition, social networking and the World Wide Web are also changing the way the influence of a researcher and organisations are profiled. As a consequence considering the influence of infometrics more broadly is considered [6].

## 2. Methods

The electronic databases CINAHL, Medline, and Scopus were interrogated using the search terms including *bibliometrics*, *nursing*, and *midwifery*. The reference lists of retrieved articles and Internet sources and social media platforms were also examined. Initial search results yielded 367 articles. Following review of titles and abstracts, 167 articles were identified as providing information to address the aims of the review. These articles were not only descriptive in terms of bibliometrics but also address implementation issues as well as the benefits and shortcomings of approaches. These articles were synthesized and methodological approaches and strengths and limitations of approaches identified.

## 3. Current Approaches to Bibliometrics

### 3.1. Impact Factors

Journal impact factors (IFs) are the measure of the frequency in which an “average article” in a journal has been cited over a defined period [7]. They are calculated by Thomson-Reuters' Institute of Scientific Information (ISI) and published each June in Journal Citations Reports. Since their inception in 1955, improvements have been made, including showing the 5-year IF and increasing the number of non-English journals included in the analysis. Data are also available for ranking the Immediacy Index of articles, which measures the number of times an article was cited in the year in which it was published [8].

However, impact factors have been subject to ongoing criticism by academics and scholars for both methodological and procedural imperfections. There is also debate about how IFs should be used. Whilst a higher impact factor may indicate journals that are considered to be more prestigious, it does not necessarily reflect the quality or impact of an individual article or researcher. Other metrics have therefore been developed to provide alternative measures to impact factors, such as the Journal Evaluation Tool [1, 9–11].

### 3.2. *h*- and *c*-Indices

Originally developed in 2005, the (Hirsch) *h*-index was developed to estimate the importance, significance, and broad impact of a researcher's cumulative research contributions [12]. The *h*-index was designed to overcome the limitations of previous measures of the quality and productivity of researchers. It is a single number reporting an author's papers that have at least the equivalent number of citations [13]. For example, a researcher with an *h*-index of 5 means that they have published at least 5 papers that have been cited 5 times or more. To obtain a high *h*-index, a researcher needs to be productive (quantity), but these papers also need to be highly cited (quality). This is likely driving individuals to publish in open access journals.

Papers can be cited for many reasons, such as proposing contentious positions; other than being of high quality and with the *h*-index, the quality of journals is not considered. Hirsch openly acknowledges that a single number cannot truly reflect the multifaceted profile of an individual author, as well as other limitations of the *h*-index [12], such as duration of publishing. An important consideration when using the *h*-index is that stage of career is a factor, and so one limitation of the *h*-index is that more junior researchers are inevitably going to have a lower *h*-index. Several recent studies have quantified the *h*-index for leading nurse academics and researchers in Canada, the United Kingdom, and Australia. These findings show significant diversity in *h*-indices of nurse researchers from these different countries and reported scores between 4 and 26 [13–15].

Similar to the Immediacy Index for journals, the *c*-index reports the number of articles that have been cited more than once by other researchers in the most recent calendar year and therefore provides information about the current research impact of an article. The *c*-index has been proposed as an addition to the *h*-index [16].

### 3.3. The Performance of Nursing and Midwifery

A number of studies have been undertaken tracking the increased performance of both nursing journals and individual researchers. Wilkes and Jackson analysed a total of 530 articles from five Australian and five USA and UK journals and found an increase in output from the period of prior analyses in 2000 [17]. Publication analyses of Canadian publications [13] and UK [15] and Australian nurses have been undertaken. Hack and colleagues observed that nurses with an *h*-index of 10–14 indicated an excellent publication record [13]. Thompson and Clark cite the five top bad reasons nurses do not publish in high impact journals and among these are the need to influence nurse clinicians and reach a particular audience [18]. They argue that ignoring bibliometrics is folly and we should strive to publish in journals that are highly influential across disciplines. Discrete specialties have also undertaken reviews demonstrating trends in citation rates and trends using particular publishing patterns [19].

The notion of impact is also not easy to measure, a case for many disciplines. In health care, measures of impact can be constructed as being of scholarly impact (where citation measures are very useful) or impact on clinical practice [20]. This latter aspect of impact may be of greater importance in a practice-based discipline, but it can be difficult to provide evidence on this, as there are so many factors that influence uptake of research findings into health care. This in itself is an area of increasing research and scholarly interest [21].

### 3.4. New Approaches to Measuring Performance

In order to deal with the complexity of citation impact analysis, a range of approaches have been introduced including percentile rank scores, as indicators of relative performance [22]. The Integrated Impact Indicator (I3) has been suggested as a congruous indicator of absolute performance overcoming other aspects of measurement issues [22].

Globally, the use of the Internet is increasing exponentially [23]. Web 2.0 allows Internet users to independently create and publish content rapidly. Never before has it been easier for academics to provide rapid responses to media requests and publically provide opinion and commentary on both current affairs and scientific findings. The influence of social media is truly changing the academic publishing landscape. So much that there has been increased recognition for measures of scholarly impact to be drawn from Web 2.0 data [24].

The World Wide Web has not only revolutionized how information is gathered, stored, and shared but also provided a mechanism of measuring access to information. The current debate and discussion of the online publishing forum and the importance of access to information and challenging traditional gatekeepers to knowledge are a critical consideration [25]. Moreover, the use of blogs as scholarly sources has been introduced [26]. This increases the view of assessing performance than merely moving traditional bibliometrics to a more germane view of infometrics [6].

### 3.5. Webometrics

Webometrics refers to the quantitative analysis of activity on the World Wide Web, such as downloads, which draws on infometric methods [27, 28]. Webometrics recognises that the Internet is a repository for a vast number of documents and a powerful vehicle for knowledge dissemination and access [29, 30]. Ranking involves measuring the volume, visibility, and impact of web pages published by universities, with special emphasis on scientific output (refereed papers, conference contributions, preprints, monographs, theses, and reports), but also examines other materials (courseware, seminars or workshop documentations, digital libraries, databases, multimedia, and personal pages and blogs) and the general information on the institution, their departments, research groups or supporting services, and people working or attending courses. Ranking can be undertaken using a number of approaches.

Thus it can be seen that measurement of scholarship and impact can occur using a range of metrics. Within both traditional and evolving approaches it is useful to review the performance of nursing and midwifery according to established measures.

### 3.6. Innovations in Bibliometrics

Criticisms of traditional approaches to bibliometrics, such as impact factors and citations, included a perceived disadvantage for certain disciplines. However, conversely it can be said that citations in nursing and midwifery, like other areas of health, can accumulate relatively quickly. This is attributable to the large number of journals, the volume of research being conducted, and also the rapidly changing nature of the field and the increasing representation of nurses and midwives in research. This is particularly so when compared to disciplines with fewer journals or disciplines in which change or evidence of impact is achieved rather more slowly, such as in mathematics, for example.

### 3.7. Journal Evaluation Tool

Sponsored by the Council of Deans of Nursing and Midwifery in Australia and New Zealand, the Journal Evaluation Tool (JET) rates journals according to four quality band scores [9]. The JET involves peer ranking of journals and was designed as a strategy to overcome some of the traditional metrics of impact factors which have been said to disadvantage some groups such as nurses, midwives, and general practitioners. One significant drawback of the JET tool is that it has no standing outside of Australia and New Zealand. Clearly, researchers and scholars need to operate in an international environment and so need to be mindful of internationally (rather than locally) recognised measures.

### 3.8. Web 2.0 and Social Media

Twitter is a microblogging platform that allows users to “tweet” text of up to 140 characters to users and is publically available to anyone with online access. Twitter is commonly used as an online communication platform for personal communications; however it is rapidly becoming used for work related purposes, particularly scholarly communication, as a method of sharing and disseminating information which is central to the work of an academic [31]. Recently, there has been rapid growth in the uptake of Twitter by nursing and midwifery academics to network, share ideas and common interests, and promote their scientific findings.

### 3.9. Twitter Citation, Twimpact Factor, and Twindex

A study conducted by Eysenbach [32] investigated the predictive ability of Twitter for “citations,” defined as “*direct or indirect links from a tweet to a peer-reviewed scholarly article online*” [33]. Eysenbach developed a metric he termed as the “twimpact” factor and suggested that this may be useful and timely for measuring the uptake of research findings and for filtering research findings resonating with the public in real time [32].

The twimpact (tw*n*) factor is a novel metric for immediate impact in social media, defined by the cumulative number of tweetations within *n* days after publications (e.g., tw7 means totals number of tweetations after *n* = 7 days). Here tweetations are URL mentions if we apply this to other social media platforms [32].

The twindex is a metric ranging from 0 to 100 indicating the relative standing of an article compared to other articles. The twindex7 of specific articles is the rank percentile of an article when all articles are ranked by the twimpact factor tw7. For example, if an article has the highest twimpact factor tw7 among its comparator articles, it has a twindex of 100. In Eysenbach's seminal work on the ability of tweets to predict citations, twindex articles with >75 often turned out to be the most cited [32].

Whilst the study identified that the buzz of the blogosphere is measurable, many limitations are also noted including the fundamental observation that the number of hits is a metric of success. The authors also identified that correlation is not causation, and it is difficult to decide whether additional citations are a results of the social media buzz or whether it is the underlying quality of the article or news trustworthiness that drives both the buzz and the citations—it is most likely a combination of both [32]. This novel study warrants further investigation into the sensitivity and specificity of such metrics to predict citations, particularly in nursing and midwifery.

### 3.10. Forecasting Popularity in Social Media

A preliminary study conducted by Yan and Kaziunas identified that merely measuring the dominance of an academic institution in Twitter is not a comprehensive measure of the true worth of a tweet. Additionally, users in academic institutions are more likely to derive value from the quality of the content. Results of this study are limited due to the small sample size [34]. Bandari and colleagues [35] suggested that one of the most significant predictors of popularity in social media was the news source of the article and that this is supported by the reality that readers are often likely to be influenced by the news source disseminating the article [35]. While popularity or number of hits or tweets may not be directly related to quality and impact, one could extrapolate that a hit or tweet does indicate interest at least, with the possibility that the article will be read and may be used in some way to either inform clinical practice or scholarly work.

### 3.11. Klout!, PeerIndex, and Kred

A range of online services, such as Klout!, PeerIndex, and Kred, attempt to measure influence in social media using various (undisclosed) algorithms and metrics; all are available free of charge. Klout! (http://www.klout.com/) uses 35 variables to compile “influence” scores, including the number of active followers a user has on Twitter, number of responses or retweets, and how influential the audience is. A higher Klout score indicates a stronger influence of the individual on the social media community [36]. A Klout score begins at 40.

Similarly, PeerIndex (http://www.peerindex.com/) calculates a score that is a relative measure of a user's online authority, reflecting the impact of a user's online activities and the extent to which they have built up social and reputational capital on the Internet [37]. There are three components to a PeerIndex score: authority, audience, and activity. Authority is the measure of trust, which calculates how much other users reply on recommendations and opinions. Audience is an indication of a user's reach, accounting for the relative size of a user's audience. Activity is a measure of how much the user does that is related to the topic communities that the user is part of [37]. Lastly, Kred (http://www.kred.com/) measures influence of and outreach to a user's social communities in real time [38]. Influence scores range from 1 to 1000, where influence is measured by the ability of the user to persuade others to take action such a retweets or replies on Twitter or Facebook “likes” or “shares.” Outreach points are combined into levels. Kred scans the Twittersphere for trending topics by communities and looks through the list of followers to find communities to identify content user's followers have not published [38]. Of these three online influence calculators, Kred claims to have the most transparent measures of influence and outreach in social media, through the generation of unique scores for every domain of expertise [38].

These are a taste of the tools available to measure and examine impact in the social media and online world. Others exist including Twitter Grader and Social Bro. The main disadvantage with such tools is that they merely measure activity and engagement. However, central to an academic's work are credibility and peer review.

## 4. Discussion

In reducing impact to a quantitative, numerical score, it could be argued that bibliometrics are highly reductionistic and, when viewed in isolation, are not representative of a researcher's performance or capacity. In taking this view, one would view bibliometric measures as only one aspect of performance upon which academic/scientific standing can be judged. However, bibliometrics have a high utility, and this is likely to continue because in pragmatic terms they represent a relatively simple, notwithstanding any weaknesses, and accurate data source.

As we have suggested earlier in this paper, there are various sources of bibliometric data, each with their strengths and limitations. What is needed is broad agreement on the most useful indices. Though bibliometric measures are best applied in a combination of methods of impact and esteem, other measures of these are far more difficult to quantify. Measures of esteem are defined as the recognition of researchers by peers, for their achievements, leadership, and contribution to a field of research [39]. This is most easily demonstrated through the award of prizes and prestigious invitations such as international keynote addresses, editorial roles, or membership of peak bodies. However, these measures are also controversial and in some quarters such activities may be viewed as being indicative of an individual's personal network, rather than real evidence of wider professional standing or esteem. Increasingly other measures of influence, using criteria of social media, are identified.

## 5. Where to from Here? 

### 5.1. Open Access

Expanding access to research findings is paramount for scientific progress. Debate continues concerning the public's right to access taxpayer, publically funded research findings. The National Institute of Health (NIH) in the Unites States now mandates that published results of all NIH funded research are archived in the National Library of Medicine's PubMed Central, to be available to the public no later than 12 months after publication [40]. Other public funding bodies around the world have begun to use a similar approach. Increasing access to scientific work has seen many institutions creating open access repositories of articles published by their staff, in a manner consistent with relevant copyright laws. This normally involves making a copy of the electronic version of their final, peer-reviewed manuscript available. Finding a model that is acceptable to the scientific community, funding agencies, governments, and publishers is however proving difficult.

Open access journals are becoming increasingly in evidence, and their presence presents new options for scholars seeking to disseminate their work. Their open access status is a major advantage. The fact that these papers are widely (and freely) available should assist in ensuring optimal citations. A limitation of open access for readers however means that many of these journals charge authors a publishing fee. This fee is applied in addition to the usual stringent peer review process. A recent survey of peer-reviewed, English-language open access nursing journals (*n* = 11) reported that only five (of the 11 journals) had *h*-indices on Scopus and five had a listed JIF (range: 0.21–2.00) and that publication fees ranged from zero (*n* = 4) to AU$1945 [41].

### 5.2. The Individual or the Organisation?

Ranking universities as a single entity may not be the most appropriate way to identify where the best discipline-based research is performed, and it is unlikely that any single university will excel in all disciplinary areas. Therefore the ranking of disciplines (as independent entities) may have some broader utility, although an unintended consequence of this may be the stifling of interdisciplinary research, clearly an important goal within constrained funding environments.

## 6. Conclusions

Nurse and midwife researchers can no longer choose to avoid the process and politics of bibliometrics or measure of impact. The productivity and quality of research produced by individual researchers, research groups, and universities are an important metric of their success and contribution to the productivity of the economy. Despite the criticism and acknowledged weaknesses of bibliometric measures they form a vital function of this equation. Like most measures these indices should be scrutinised for validity and fitness for purpose. This will require ongoing development and evaluation on a regular basis as new opportunities emerge, particularly though online media.
